# Novel polymeric micelles for insect pest control: encapsulation of essential oil monoterpenes inside a triblock copolymer shell for head lice control

**DOI:** 10.7717/peerj.3171

**Published:** 2017-04-20

**Authors:** Alejandro Lucia, Ariel Ceferino Toloza, Eduardo Guzmán, Francisco Ortega, Ramón G. Rubio

**Affiliations:** 1 Centro de Investigaciones de Plagas e Insecticidas (UNIDEF-CONICET), Villa Martelli, Buenos Aires, Argentina; 2 Departamento de Química Física, Universidad Complutense de Madrid, Madrid, España; 3 Instituto Pluridisciplinar, Universidad Complutense de Madrid, Madrid, España

**Keywords:** Encapsulation, Monoterpenes, Head lice, Polymer- based nanomicelles, Poloxamers

## Abstract

**Background:**

Essential oil components (EOCs) are molecules with interesting application in pest control, these have been evaluated against different insect pest from more than 100 years, but their practical use is rather limited. Thus, the enhancement of their bioavailability and manageability due to their dispersion in water can open new perspective for the preparation of formulations for the control of insect pest. In this work, we studied the encapsulation of different monoterpenes in a poloxamer shell in order to prepare aqueous formulations that can be used for the development of platforms used in pest control.

**Methods:**

Micellar systems containing a 5 wt% of poloxamer 407 and 1.25 wt% of the different monoterpenes were prepared. Dynamic Light Scattering (DLS) experiments were carried out to characterize the dispersion of the EOCs in water. The pediculicidal activity of these micellar systems was tested on head lice using an *ex vivo* immersion test.

**Results:**

The poloxamers allowed the dispersion of EOCs in water due to their encapsulation inside the hydrophobic core of the copolymer micelles. From this study, we concluded that it is possible to make stable micellar systems containing water (>90 wt%), 1.25 wt% of different monoterpenes and a highly safe polymer (5wt% Poloxamer 407). These formulations were effective against head lice with mortality ranging from 30 to 60%, being the most effective emulsions those containing linalool, 1,8-cineole, *α*-terpineol, thymol, eugenol, geraniol and nonyl alcohol which lead to mortalities above 50%.

**Discussion:**

Since these systems showed good pediculicidal activity and high physicochemical stability, they could be a new route for the green fabrication of biocompatible and biosustainable insecticide formulations.

## Introduction

Essential oils are a special group of chemical compounds belonging to the natural products, which are in general highly volatile substances obtained from a wide variety of sources (Weinzieri, Palevitch & Craker, 1994). Generally, most of the essential oils are obtained using steam-distillation of vegetal tissues either from wild or cultivated plants, and consist in complex mixtures of hydrocarbons, mainly terpenes and sesquiterpenes, and oxygenated compounds (alcohols, esters, ethers, aldehydes, ketones, lactones, phenols and phenol ethers) (Guenther, 1972).

The different components of essential oils have different physico-chemical properties. This becomes them in interesting molecules for designing formulations with application in pest control. This is due in part because most of the phytochemicals act as general toxic agents against insects in different life stages. In addition, they may interfere with growth, reproduction of the insects, or act on their olfactory receptors, inducing attraction or repellency effects (Sukumar, Perich & Boombar, 1991; Isman, 2000; Gonzalez-Coloma et al., 2011). Essential oils and their pure components possess an extraordinary bioactivity, being reported their use against a wide range of insect pest, e.g., *Aedes aegypti* (Lucia et al., 2007; Lucia et al., 2008; Lucia et al., 2009; Lucia et al., 2012; Pavela, 2015), *Pediculus humanus capitis* (Toloza et al., 2008; Toloza, Vassena & Picollo, 2008), *Tribolium castaneum* and *Sitophilus oryzae* (Lee, Peterson & Coats, 2003; Lopez, Contreras & Villalobos, 2008; Stefanazzi, Stadler & Ferrero, 2011) *Trichoplusia ni* (Tak, Jovel & Isman, 2015; Tak & Isman, 2015). Essential oils have even been evaluated against different insect pest from more than 100 years; their practical use is rather limited.

The research on pesticide formulation has undergone a strong increase during the last twenty years, leading to new pesticide formulations developed by different manufacturing processes (Schwartz et al., 2003; Venkatesan, Manavalan & Valliappan, 2009; Rao, Deepthi & Chowdary, 2009; Bauer & Baumann, 2015). The main problem associated with this development is the optimization of the effectiveness of the formulation, as well as its safety, handling, and storage conditions. Therefore, the pesticide compounds in their “raw” or unformulated state are not usually suitable for pest control, since these concentrated chemicals and active ingredients might not mix well with water, may be chemically unstable, and may be difficult to handle and transport. For these reasons, manufacturers add inert substances, such as clays and solvents in order to improve their characteristics (Fishel, 2016). In this way, the challenge in the present and future is the design and development of well sketched vectors, with a well-established and known chemical composition as well as physical properties, for loading and controlled delivery of active compounds. In the last years, the research activity of many groups has been focused on the development of new efficient platforms which overcome the main limitations associated with traditional ones (McClements, 2015).

Recently, several attempts have been carried out to manufacture effective vectors based in natural products for their application in different technological areas, e.g., *surfactant free emulsions* (Drapeau et al., 2009; Marcus et al., 2014; Lucia et al., in press), nanocrystals or nanoparticles (Murugan et al., 2016), nanoemulsions (Sakulku et al., 2009), vesicles, liposomes and micelles (Bilia et al., 2015), gels (Barradas et al., 2013), Pickering emulsions (Adelmann, Binks & Mezzenga, 2012), microspheres or micropearls (Soliman et al., 2013), creams (Oyedele et al., 2002), Microcapsules (Leimann et al., 2009; Karr, Speaker & Kasting, 2012; Banerjee et al., 2013; Pavela, 2016). Polymer based capsules have previously been tested as carriers for carvacrol, thymol and eugenol (Sajomsang et al., 2012; Higueras, López-Carballo & Cerisuelo, 2013).

The combination of essential oils with an adjuvant can provide the foundation for the fabrication of new aqueous formulations with enhanced efficiency and environmentally friendly. Adjuvants formulation to enhance mixing or application, or to improve pesticide activity (Fishel, 2016). Block copolymers with surfactant character (poloxamer family) can be a good example of adjuvant agent, which can act as antifoaming and wetting agents, dispersants, thickeners, and emulsifiers. The poloxamer 407 is an amphiphilic molecule, that above a critical temperature and concentration; self-aggregate in aqueous solutions to form spherical micelles with a hydrophobic core of polyoxypropylene (poly (propylene oxide)) surrounded by hydrophilic corona of polyoxyethylene (poly (ethylene oxide)) (Yardimci et al., 2005; Basak & Bandyopadhyay, 2013). This has led to the application of their micelles or gels for the development of different drug carrier systems (Alexandridis, Zhou & Khan, 1996; Ivanova, Lindman & Alexandridis, 2000; Bivas-Benita et al., 2004; Pepic, Jalsenjak & Jalsenjak, 2004; Escobar-Chávez et al., 2006). It is worth mentioning that the capacity of the drug encapsulation and release can be easily controlled in this systems (Sharma & Bhatia, 2004; Ruel-Gariepy & Leroux, 2004; O’Callaghan, 2010). Poloxamers have been used in the preparation of drug carries allowing for a decrease or delay of the permeation of DEET (insect repellent) through the skin (Batrakova & Kabanov, 2008; Barradas et al., 2013).

The aim of this work was the encapsulation of different essential oil compounds (EOCs) in a poloxamer shell in order to prepare aqueous formulations that can be used for the development of platforms used in pest control. It is expected that the formulations obtained can present a high bioactivity against insect pest, mainly *Pediculus humanus capitis*, enhancing the distribution of the active ingredient. In this work, the formation of polymer shells protecting EOCs drops is tested as an effective methodology to prepare new bioactive formulations for pest control.

## Materials and Methods

### Chemicals

The different monoterpenoids used in the present study were Eugenol (purity 99%), 1,8-Cineole (purity 99%), Geraniol (purity 98%), Linalool (purity 97%), Carvacrol (purity 98%), α-terpineol (purity > 96%), Citronellol (purity > 95%), Nonyl alcohol (purity 99%), Thymol (purity 99.5%) and Menthol (purity 99%). All were purchased from Sigma-Aldrich and used without further purification. For sake of simplicity, the different monoterpenoids will be referred as Essential Oil Compounds (EOCs) in the following.

Poloxamer 407, also known as Pluronic^®^ F-127, is a triblock copolymer formed for two lateral blocks of poly(ethylene oxide) (PEO) and a central block of poly(propylene oxide) (PPO), being the number of monomer about 101 and 56 for PEO and PPO blocks, respectively. This leads to an average formula [PEO_101_PPO_56_PEO_101_] and an average molecular weight of 12.5 kDa. Poloxamer 407 was also purchased from Sigma-Aldrich (Germany) and used without further purification.

All solutions were prepared by weight using ultrapure deionized water (Milli-Q quality) obtained by a multicartridge purification system (Young lin 370 Series; Young Lin, Angyang, South Korea) presenting a resistivity higher than 18 MΩ cm and a total organic content lower than 6 ppm.

### Sample preparation

**Figure 1:**
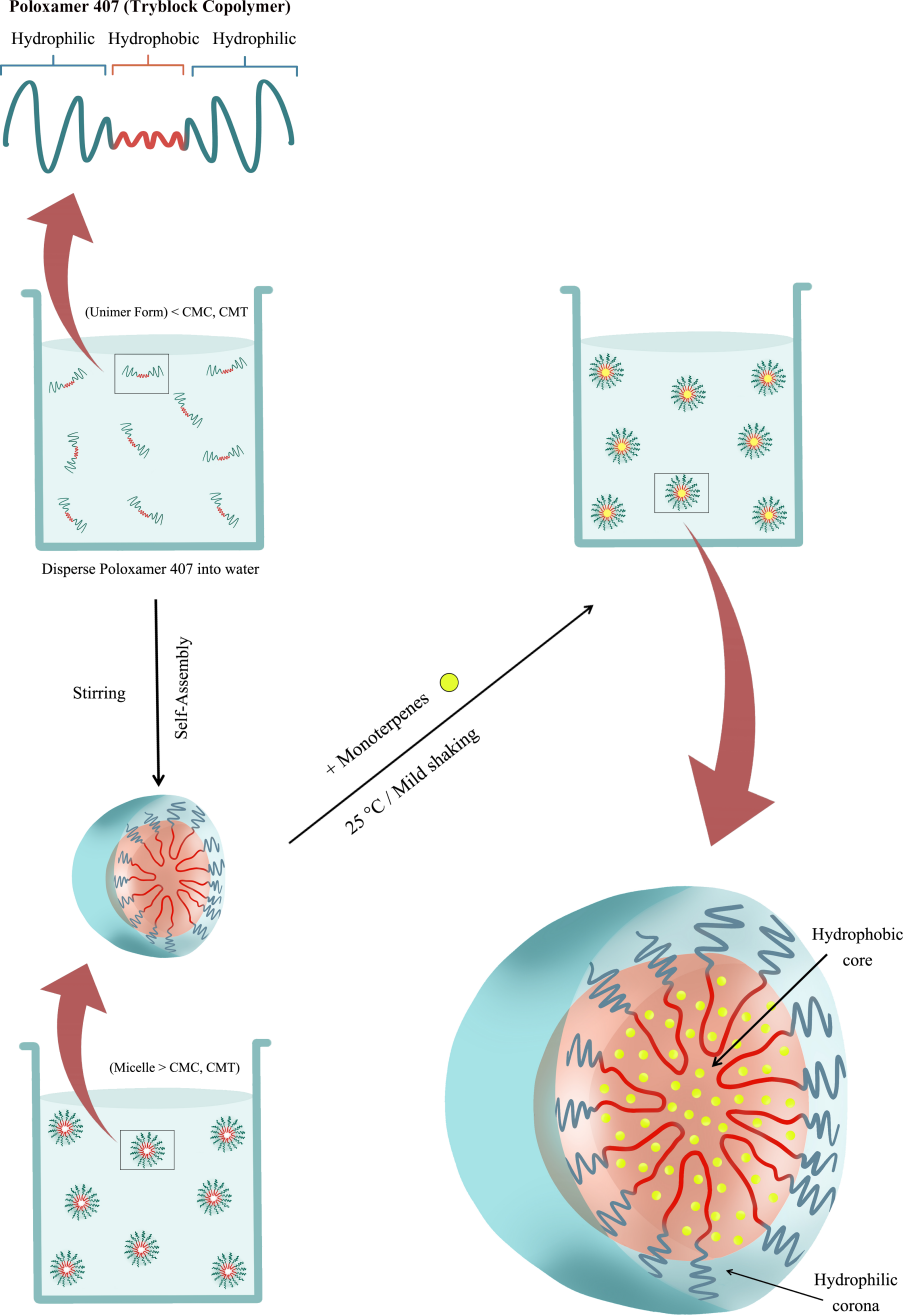
Scheme of the EOCs encapsulation process by solubilization inside the hydrophobic region of Poloxamer 407 micelles. CMC, Critical micelle concentration; CMT, Critical micelle temperature.

Emulsions were prepared in tubular glass vials (10 ml). For this purpose, poloxamer 407 solutions, prepared by stirring, with concentration 10.5 wt% in Milli-Q water were added in the vial. These solutions had a concentration higher than the CMC of poloxamer 407 at the temperature used in this work (25 °C). Once poloxamer was in the vial, the different EOCs were added up to a final concentration of 2.5 wt% and the mixtures were homogenised by mild shaking Fig. 1. The process led to opalescent dispersions which were diluted in a 1:1 ratio using Milli-Q water. Thus, formulations containing a 5 wt% of poloxamer 407 and 1.25 wt% of the different monoterpenes were prepared. The base formulation consisted of 5wt% polaxamer 407 aqueous solution. During all the process, the temperature remained constant at 25 °C. Once prepared, solutions were stored at at room temperature (19–23 °C).

### Dynamic Light Scattering (DLS)

Dynamic Light Scattering (DLS) experiments were carried out using a Zetasizer Nano ZS (Malvern Instruments Ltd., Worcestshire, UK). All the DLS measurements were performed at 25 °C using as radiation the red line (wavelength, λ = 632 nm) of a He-Ne laser in a quasi-backscattering configuration (scattering angle, θ = 173°). In a previous step to the measurements, samples were filtered in a clean-room using a Nylon membrane with a pore diameter of 0.45 µm (Millex; Millipore, Billerica, MA, USA) in order to remove possible dust particles in the samples. Once, the samples were filtered and transferred to the quartz cell used for the measurement (Hellma^®^6030-OG Model; Hellma, Jena, Germany).

In DLS experiments, the normalized intensity or second-order autocorrelation function, *g*^(2)^(*q*, *t*) is obtained. This is related to the field or first-order autocorrelation function, *g*^(1)^(*q*, *t*), through the Siegert relationship

(1) \begin{eqnarray*}{g}^{(2)}(q,t)-1=\beta {|}{g}^{(1)}(q,t){{|}}^{2}\end{eqnarray*}

where *t* is the time, *q* = (4*πn*∕*λ*)sin(*θ*∕2) is the wavevector, and *n* the solution refractive index. This latter was assumed to be close to that of the continuous phase (pure water, *n* = 1.33). In Eq. (1), β is an optical coherence factor and is generally found to be close to 1, except for those cases in which the scattered intensity is relatively low. This decrease of the optical coherence factor can be ascribed either to the small size of the scatterers, low concentration or poor refractive index contrast between the scatterers and the solvent. Three replicas of each measurement were carried out, being the deviation between the different replicas less than 1%.

### Pediculus humanus capitits (Head lice)

Head lice were collected from infested children 6–13 years old, using a fine toothed antilouse metallic comb. Children were examined during the period July 16 to November 9, 2015. A total of 880 lice were obtained from three elementary schools located in Buenos Aires, where a topical method indicated high resistance levels to permethrin (71.42, 35.37 and 33.33, respectively) (Toloza, 2010). All the studied schools were Argentinean Government owned and non-fee-paying. Only pupils whose parents had given informed consent for participation were examined. The freedom to refuse to participate in the research was clearly established in each case. As the present research was not an interventional study as stated by the Argentinean regulations, acceptance of the protocol by an ethical commission was not required at the time of this research work. Once collected, head lice were transported to the laboratory according to the protocol developed by Picollo et al. (1998). The protocol for lice collection was approved by an ad hoc committee belonging to Centro de Investigaciones de Plagas e Insecticidas (CONICET-UNIDEF), Buenos Aires, Argentina. Adults and third instar nymphs were selected at the laboratory for the bioassays (Picollo et al., 1998). After collection, insects were examined carefully under OLIMPUS SZ4045 stereomicroscope and any damage lice were discarded. Then, they were transferred to an environmental chamber (Lab-Line instruments, Melrose Park, IL, USA) at 18 ± 0.5°C, 70–80  ± 1% relative humidity (RH) in the dark until they were tested. The period between head louse collection and the start of the experiments was no longer than 2 h.

### Immersion test against Pediculus humanus capitits

The adulticidal and third-stage nymph activity was assessed using an *ex vivo* immersion test (Gallardo & Mougabure-Cueto, 2012). The *ex vivo* test consisted in the fully immersion of different batches of at least 10–15 lice deposited in a lid of a Petri dish (diameter 5.5 cm) during 5 min in 2 mL of the different prepared emulsions. Once the exposure period was finished, the insects were placed onto a metallic mesh and rinsed with 100 mL of water. Then, lice were transferred onto a Whatman^®^ qualitative filter paper (grade 1; Whatman, Marlborough, MA, USA) moistened with 0.5 mL of water, and placed in the bottom of a plastic Petri dish. During the studied period, Petri dish containing head lice were enclosed in plastic containers where distilled water (high RH) was added (with no contact with the animals). This containers were kept inside an environmental chamber (Ambi-Hi-Low Lab-line, Iowa, USA) set at 18 ±0.5°C and 70–80 ± 1% relative humidity (RH) in the dark (Gallardo, Mougabure-Cueto & Picollo, 2009). Control consisted of healthy living lice (*n* = 10–15 per replicate per treatment) placed in the lid of a Petri dish and immersed into distilled water following the same procedures as the experimental group.

Exposed lice to formulations or control were observed under stereomicroscope by the same observer at room temperature (19– 24 °C) during 7 h and at 18 h in order to find any lethal activity of the formulations. The time for measurement of affected lice in a bioassay should consider the recovering of insects after the exposure treatment (Combescot-Lang et al., 2015). Thus, we considered 7 h after treatment as the observational reference period reliable to estimate pediculicidal effectiveness. However, after this period, it is still possible to detect lice recovering. Thus, lice were also observed at 18 h since it was estimated as the optimal period for comparative bioassays of human lice (Gallardo, Mougabure-Cueto & Picollo, 2009).

Criterion of affected insects was according to Combescot-Lang et al. (2015). Briefly, a louse was considered alive if it showed no symptoms or some abnormal movements and having difficulties turning over. On the other hand, it was considered dead if it remained on its back showing no external or internal movements, except for slight contraction of the digestive tube. A minimum of at least three-five replicates (*N* = 70) of the toxicity assays were carried out for each formulation and control.

### Statistical analysis

The percentage of insects affected or their degree of mortality were determined and transformed to arcsine square-root values for analysis of variance (ANOVA). The values obtained were compared and separated by the Tukey test (StatSoft Inc., v. 7; Tulsa, OK, USA). All statistical tests were performed with α = 0.05 for significance of statistical tests.

## Results

### Dispersion characterization

**Figure 2:**
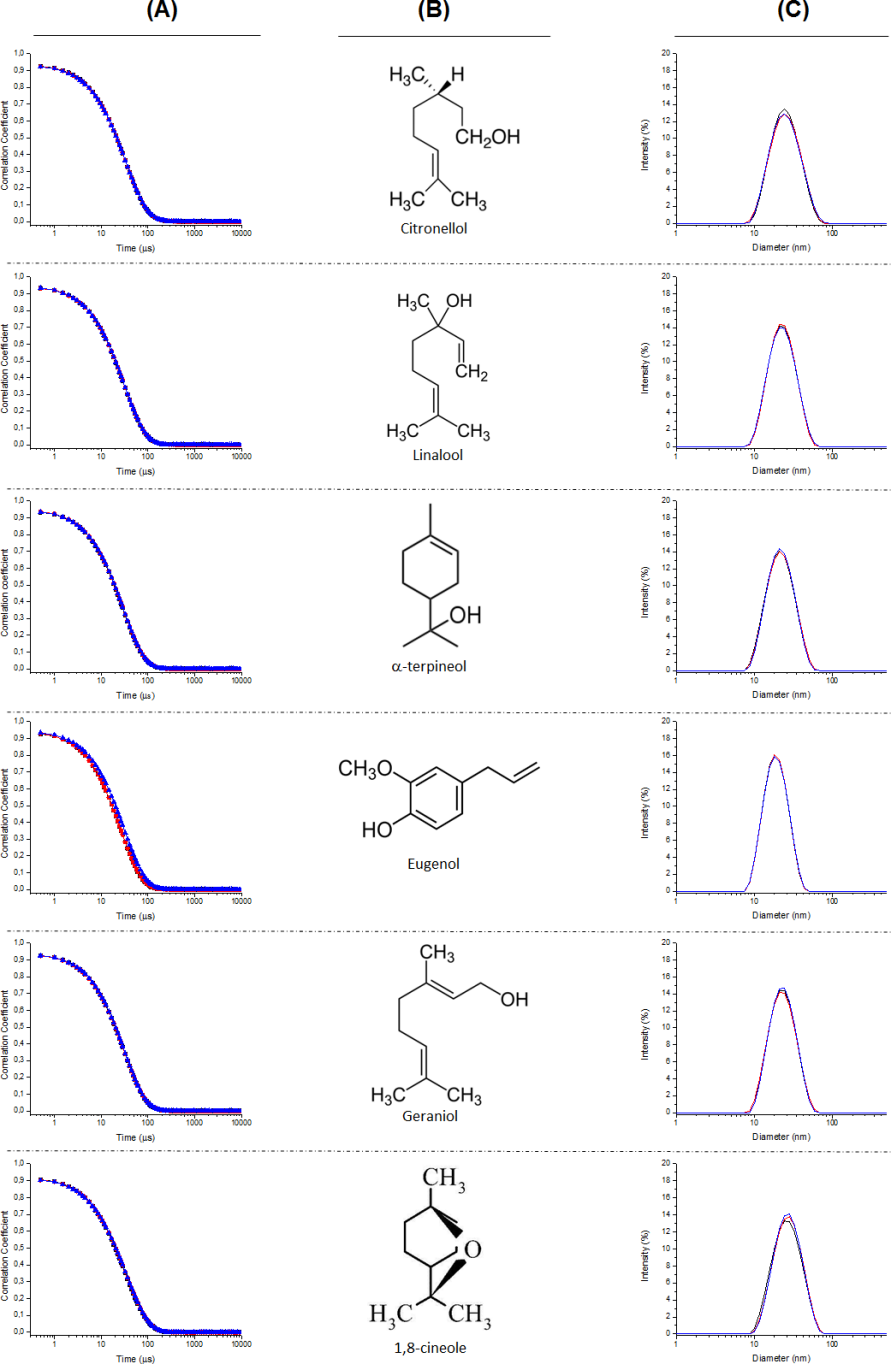
Auto-correlation functions (column A), chemical structure of the EOCs used (column B), and representative average size distributions (column C) of the polymeric micelles composed of Poloxamer 407 and EOCs using DLS for monomodal samples.

**Figure 3:**
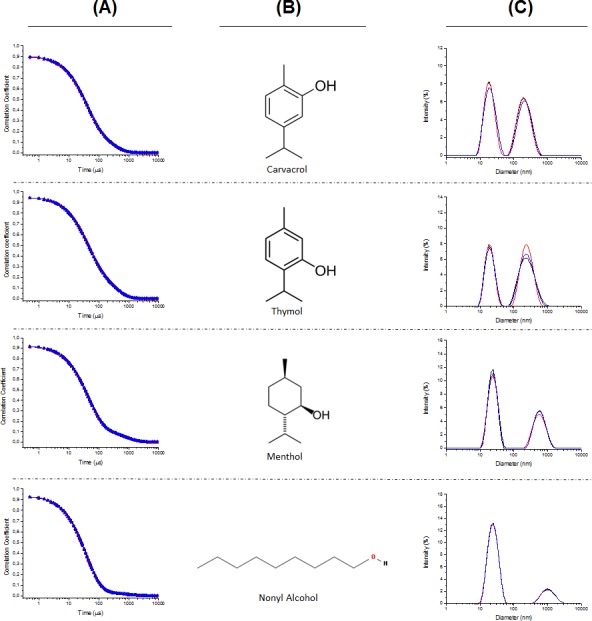
Auto-correlation functions (column A), chemical structure of the EOCs used (column B), and representative average size distributions (column C) of the polymeric micelles composed of Poloxamer 407 and EOCs using DLS for bimodal samples.

The first step of the study carried out was the characterization of the dispersion of the EOCs in water assisted by the addition of poloxamer 407. The Dynamic Light Scattering (DLS) experiments shows the representative intensity auto-correlation functions and average size distributions of the aggregates obtained for different formulations. The formulations containing six of the EOCs (geraniol, citronellol, 1,8-cineole, linalool, α-terpineol and eugenol) showed a clearly monomodal decay (Fig. 2), whereas the intensity auto-correlation functions for the other four formulations (nonyl alcohol, thymol, menthol and carvacrol) had a clear bimodal character (Fig. 3). Table 1 summarizes the average diameter for monomodal samples and relative importance of the different components of the size distribution for bimodal samples.

### Bioactivity assays

The above characterized formulations were tested against permethrin-resistant head lice from Argentina. The results showed that the formulations prepared present a significant effect when they are used against lice. According to the results obtained in this work, the most effective EOC emulsion at either 7 or 18 h was linalool with a mortality (%) of 61, followed by 1,8-cineole (56), α-terpineol (55), thymol (53), eugenol (51,8), geraniol (51,3), nonyl alcohol (50), menthol (43,3), citronellol (39) and carvacrol (28) (Table 2). Moreover, there were no significant differences in the mean of the affected lice at both 7 and 18 h. Thus, treated lice at 7 h did not recovered during next hours.

**Table 1:** Polydispersity index (PDI), average diameter for monomodal samples and relative importance of the different components of the size distribution for bimodal samples. Notice the error corresponds to the standard deviation of different measurements.

EOC Nanoemulsion	PDI	Average Diameter –Size distribution (nm)	
Eugenol^a^	0.090 ± 0.002	17.8 ± 0.1	
Eucalyptol^a^	0.200 ± 0.002	22.2 ± 0.4	
Geraniol^a^	0.120 ± 0.001	24.3 ± 0.1	
Linalool^a^	0.130 ± 0.002	20.9 ± 0.1	
*α* −terpineol^a^	0.130 ± 0.006	19.8 ± 0.1	
Citronellol^a^	0.150 ± 0.002	22.6 ± 0.1	
Nonyl alcohol^b^	0.350 ± 0.007	Peak 1: 25.0 ± 0.6 (82%)	Peak 2: 1106 ± 3.6 (18%)
Thymol^b^	0.830 ± 0.008	Peak 1: 19.9 ± 1.2 (43%)	Peak 2: 270 ± 2.5 (57%)
Menthol^b^	0.600 ± 0.004	Peak 1: 24.2 ± 0.7 (62%)	Peak 2: 612 ± 1.2 (38%)
Carvacrol^b^	0.520 ± 0.003	Peak 1: 20.0 ± 0.9 (51%)	Peak 2: 216 ± 0.7 (49%)
Poloxamer 407^b^	0.179 ± 0.008	Peak 1: 5.5 ± 0.2 (10%)	Peak 2: 43 ± 1.6 (90%)

**Notes.**

Distribution type:

a Monodisperse sample.

b Polydisperse sample. Poloxamer 407: Poloxamer aqueous solutions (5 wt%).

**Table 2:** Adulticidal activity of nanoemulsions after *ex vivo* exposure.

Compounds	Affected lice (%) ± SE		
	Time (h)^a^		
	7	18	
Control	0.2 ± 1.4 a	1.54 ± 3.8 a	
Base Formulation(Poloxamer 407)	10.16 ± 11.55 a	13.16 ± 9.93 a	
Carvacrol	28.33 ± 7.63 b	30 ± 5 b	
Citronellol	40 ± 1.41 c	40.50 ± 0.57 c	
Menthol	43.33 ± 15.28 cd	46.67 ± 12.58 cd	
Nonyl alcohol	50 ± 1.41 cde	52.25 ± 2.75 cde	
Geraniol	51.25 ± 18.43 cde	53.75 ± 16.52 cde	
Eugenol	51.79 ± 8.03 cde	52.35 ± 8.06 cde	
Thymol	52.80 ± 9.44 de	55.80 ± 10.08 de	
*α*-terpineol	55 ± 12.91 de	58 ± 10.3 de	
1,8-cineole	55.75 ± 14.10 de	58.61 ± 11.73 de	
Linalool	60.67 ± 12.61 e	66.17 ± 9.02 e	

**Notes.**

a Means in same column followed by different letters are significantly different by Tukey test ( *P* < 0.05).

## Discussion

### Dispersion characterization

The interest of this encapsulation process is associated with the fact that most of the monoterpenes are lipophilic compounds insoluble in water and hence, the circulation and distribution through the louse hemolymph (open circulatory system) without an adequate vehicle may be slow and limited. Therefore, they need to be dispersed within an aqueous formulation in order to ensure their effectiveness and bioavailability.

In general, polymer micelles consisting of amphiphilic block copolymers form a hydrophobic core, in which lipophilic drugs can be physically incorporated. Hydrophilic blocks or segments generate water-friendly corona and encapsulate the hydrophobic core (Kim & Park, 2010). As a result, polymer micelles loaded with hydrophobic drugs ranges from 10 to 200 nm can be successfully solubilized in aqueous media (Kim & Park, 2010). According to Batrakova et al. (2006) there are three major methods for loading drugs into polymer micelle cores: (1) chemical conjugation, (2) physical entrapment or solubilization, and (3) polyionic complexation (e.g., ionic binding). In addition, a variety of drugs can be physically incorporated into the core of the micelles by engineering the structure of the core-forming segment. Because of that, the physical incorporation or solubilization of drugs within block copolymer micelles is generally preferred over other techniques (Batrakova et al., 2006).

The analysis of the intensity auto-correlation function points out different aspects of the formulations prepared. First, the concentration of poloxamer is high enough to assume the formation of dispersions in which the drops of EOCs are encapsulated in the hydrophobic core of poloxamer 407 aggregates, similar to micelles, thus allowing the homogenous dispersion of the EOCs in water. Second, the characteristics of the aggregates are strongly dependent on the type of the EOCs used for the formulation preparation. This is particularly evident from the analysis of the type of the intensity auto-correlation function. It is worth mentioning that there are no physico-chemical reasons allowing for an explanation of such differences in the properties.

From the analysis of the intensity auto-correlation functions, it was possible to obtain information about the size distribution of the EOCs aggregates. The analysis of the size distributions obtained from the DLS experiments confirm the aforementioned differences as function of the type of EOCs used. For those formulations presenting monomodal character, the average diameter of the aggregates is centred in values about 20 nm, independently of the nature of the EOCs. Similar values are found for the formulations showing multimodal decay but aggregates with higher sizes are also found in these systems. These latter drops present an average diameter strongly dependent on the specific EOCs used.

The results do not allow us to extract any correlation between the polydispersity of the aggregates and the nature of the EOCs used. It is worth mentioning that at least a certain chemical effect is behind the formation of multimodal samples as suggest the size distributions obtained for thymol and carvacrol, which are only differentiated by the position of the hydroxyl group. In these cases, both populations show similar importance, whereas for the other EOCs showing bimodal distributions, the aggregates with smaller size are in major proportion. Furthermore, it is worth mentioning that this possible chemical effect is also reflected in the similarities found in the size of the bigger aggregates with thymol and carvacrol.

### Bioactivity assays

Using as reference the normal mortality of non-exposed lice (control), the mortality associated with the use of the formulations containing EOCs is between five and 10 times higher than that of the control, being this latter similar (within the error bars), than that corresponding to lice exposed to the poloxamer aqueos solutions (5 wt% of Poloxamer 407). Therefore, it is possible to assume that the base containing only the triblock copolymer has no lethal effect against lice. However, the addition of monoterpenes enhances the mortality of the formulations. The mortality of adults and eggs associated with the use of EOCs has been previously observed for similar under different experimental conditions and formulations (Toloza, Vassena & Picollo, 2008; Gonzalez-Audino et al., 2011; Gallardo & Mougabure-Cueto, 2012).

No significant differences were observed among the following EOCs; linalool, 1,8-cineole, α-terpineol, thymol, eugenol, geraniol and nonyl alcohol. On the other hand, the less effective EOC formulations were those containing carvacrol, which showed significantly different effects in relation to those with the other tested compounds. However, there are no neither any clear dependence on the chemical nature of the EOCs nor on the type of aggregates (monomodal vs. bimodal). This is particularly clear from the results obtained for the two positional isomers (carvacrol and thymol) whose formulations show a significantly different biological effect (*P* < 0.001). This may be related to the higher hydrophobicity of the carvacrol than the thymol which has been previously reported for explaining the differences in the fumigant activity and reproductive inhibition of the bruchid of kidney beans *Acanthoscelides obtectus* (Regnault Roger & Hamraoui, 1995). Similarly, the chemical structure of citronellol and linalool is rather similar; however the mortality of their formulations is rather different. Whereas for citronellol mortality around 40% was found, the use of citronellol increases the mortality up to values close to 60%. This suggest that the higher hydrophobicity reduce the effectiveness of the EOCs in aqueous formulations. Similar differences for citronellol and linalool have been found against adults and eggs of head lice from Argentina by using a vapour phase test (Toloza et al., 2006; Toloza, Vassena & Picollo, 2008). This indicates that irrespectively of the exposition method, linalool has stronger lethality than citronellol against head lice. Several authors found the same differences in the lethality of linalool and citronellol against a wide variety of insect pests like the rice weevil *Sitophilus oryzae*, the red flour beetle *Tribolium castaneum*, the house fly *Musca domestica* and the German cockroach *Blatella germanica* (Lee, Peterson & Coats, 2003). It is worth mentioning that the bioactivity test does not evidence any dependence of the effectiveness of the formulations with the nature of the aggregates formed.

In a previous work, Gonzalez-Audino et al. (2011) studied the efficacy of several oxygenated monoterpenoids in hydroalcoholic lotions against head lice. This study showed that a hydroalcoholic solution containing 60% ethanol was a good diluent of a 5% of pulegone, citral, geraniol, citronellol and linalool. The mortality effect of the mentioned lotions varied from 42% to 68% and there were no significantly differences among them (*P* > 0.05). These values were similar to those found in the present work. Considering that many monoterpenes or their oxidized forms can cause allergic contact dermatitis in some sensitive persons, the decrease of the containing of EOCs up to 1.25 wt% compound must be underline as a dermatological benefit of these novel pediculicidal formulations. Water-based formulation of less than 1.5% of the EOCs may help to reduce dermal irritation in comparison with the ethanol-based formulation previously reported in literature (Gonzalez-Audino et al., 2011).

## Conclusions

The present study have showed that is possible to prepare aqueous based formulations containing small amounts of EOCs (1.25 wt%) encapsulated in the hydrophobic core of poloxamer 407 aggregates. These formulations, containing very low concentrations of EOCs, showed a considerable bioactivity against permethrin resistant *Pediculus humanus capitis* (head lice). Moreover, after twelve months all the formulations remain stable, maintaining the same appearance than as prepared formulation without any macroscopic changes reminiscent of creaming or phase separation. Furthermore, EOCs are considered safe and non-toxic product, being included in the GRAS (general regarded as safe) category by Food and Drug Authority of USA and placed in toxicity category IV for acute dermal and inhalation toxicity (Tisserand & Young, 2014), e.g., the acute oral and dermal LD_50_ of the most effective EOC (linalool) to rat is 4,858 mg/kg and 2,000 mg/kg bodyweight, respectively. Furthermore, the acute oral and dermal LD_50_ of the Poloxamer 407 is 10,000 mg/kg and >5,000 mg/kg bodyweight. These values are higher than that reported for pyrethrum (LD_50_ = 1,500 mg/kg bodyweight) (Casida & Quistad, 1995). Thus, the incorporation of these compounds into pediculicide formulations seems to be a good, safe and viable alternative for the fabrication of biocompatible and biosustainable insecticide formulations.

##  Supplemental Information

10.7717/peerj.3171/supp-1 Supplemental Information 1 Data used for running head lice mortality

## Additional Information and Declarations

### Data Availability

The following information was supplied regarding data availability: The raw data has been supplied as  Supplemental Information 1.
